# Orthostatic plasma norepinephrine level as a predictor for therapeutic response to metoprolol in children with postural tachycardia syndrome

**DOI:** 10.1186/s12967-014-0249-3

**Published:** 2014-09-10

**Authors:** Qingyou Zhang, Xia Chen, Jiawei Li, Junbao Du

**Affiliations:** Department of Pediatrics, Peking University First Hospital, Xi-An Men Street No. 1, West District, Beijing, 100034 PR China; Department of Pediatrics, Langfang Municipal People’s Hospital, Langfang, Hebei 065000 PR China; Key Laboratory of Molecular Cardiology, Ministry of Education, Beijing, 100019 PR China

**Keywords:** Norepinephrine, Postural tachycardia syndrome, Metoprolol

## Abstract

**Background:**

Postural tachycardia syndrome (POTS) is a heterogeneous disorder that creates challenges for treatment. Beta-blocker was one of the most commonly used drugs, but it is inconsistently effective. The purpose of this study is to explore whether orthostatic plasma norepinephrine level could be an indicator of therapeutic effectiveness of metoprolol for POTS in children.

**Methods:**

Twenty-seven children with POTS were enrolled in our study. They received metoprolol treatment, and their orthostatic plasma norepinephrine levels were measured by high-performance liquid chromatography method. Three months after rmetoprolol treatment, 25 patients were followed up. A receiver-operating characteristic (ROC) curve was used to explore the predictive value of orthostatic plasma norepinephrine level.

**Results:**

The symptom severity and increment of heat rate from supine position to upright of patients positively correlated with their orthostatic plasma norepinephrine level (r = 0.599, *P* < 0.001; r = 0.633, *P* <0.001, respectively). Orthostatic plasma norepinephrine level in responders to metoprolol was significantly higher than that of nonresponders (P = 0.028). A ROC curve on the predictive value of orthostatic plasma norepinephrine level showed that the area under the curve was 0.785. Using a cutoff value for orthostatic plasma norepinephrine level of 3.59 pg/ml yielded both sensitivity (76.9%) and specificity (91.7%) in predicting the efficacy of metoprolol therapy for POTS.

**Conclusion:**

Orthostatic plasma norepinephrine level of > 3.59 pg/ml was an indicator of the effectiveness of metoprolol therapy for POTS in children and adolescents.

## Background

Postural tachycardia syndrome (POTS) is a heterogeneous disorder characterized by sustained tachycardia (≥30 bpm in adults and ≥40 bpm in children) upon standing, and relief of these symptoms with recumbence. Although the underlying causes of POTS remain unclear, several mechanisms have been proposed, including sympathetic activation, hypovolemia, and partial autonomic neuropathy [1–4]. POTS is associated with a poor quality of life and significantly functional disability [5]. There is a paucity of effective therapies [1,4]. Because of the striking tachycardia that is central to this disorder, these patients are often referred to cardiologists for diagnosis and treatment. Given that excessive tachycardia is a cardinal feature of this syndrome, a logical treatment strategy would be reducing the HR with β-adrenergic blockade. β-blockers have been reported to improve symptoms in case reports and open-label studies [6–10]. Unfortunately, it is inconsistently effective [11,12]. Differences in noradrenergic activity between hyperadrenergic and other types of POTS may explain this inconsistency, and previous studies of efficacy of β-blockers in POTS subjects did not stratify participants into POTS subtypes according to their underlying causes. Hyperadrenergic subgroup with β-blockers would seem reasonable. Furthermore, β-blockers can impair exercise tolerance in healthy subjects [13]. Higher-dose of β-blocker might worsen symptoms of patients with POTS, which raises the concern that the effectiveness of β-blockers in POTS might depend on noradrenergic activity of patients with POTS. Our hypothesis is that the orthostatic plasma norepinephrine level could help guiding β-blocker therapy in the management of POTS in children.

## Methods

### Subjects

Twenty-seven children (mean age, 11 years, ranging from 6 years to 15 years) were diagnosed as POTS at the Department of Pediatrics, Peking University First Hospital. Our study referred to the following criteria [14–17]: 1) a child has a normal heart rate at supine and no evidence of any cardiovascular diseases; 2) after standing up or getting up, a child has 2 of the following symptoms: dizziness, chest distress, chest pain, headache, palpitation, pale face, amaurosis, fatigue, discomfort, or syncope; 3) these symptoms should be relieved or diminished by recumbence, and the symptoms should occur repeatedly for 1 month; 4) in addition to the symptoms of orthostatic intolerance, POTS is diagnosed with the quantitative sign of a heart rate increase of 40 beats/min or greater or a heart rate of > 120 beats/min within the first 10 min after standing during head-up test (HUT) or head-up tilt test (HUTT); simultaneously, the decrease of blood pressure should be < 20/10 mm Hg; and 5) children with other diseases that cause symptoms of autonomic nervous system (such as anemia, arrhythmia, hypertension, or endocrine disorders including pheochromocytoma or hyperthyroidism) as well as cardiogenic or neurogenic diseases that would induce syncope were excluded. The study has been conducted according to the principles expressed in the Declaration of Helsinki. The written informed consent had been obtained from each individual and their parents and the study protocol was approved by the Ethics Committee of Peking University First Hospital (the approval number: 2012 [525]).

### HUTT protocol

All patients received tilt table testing. All examinations occurred between 8:00 AM and 10:00 AM in a quiet and bland light room at a comfortable constant ambient temperature (22°C, 50% to 55% humidity). Patients were secured to a tilt table, and HR and BP were monitored for 10 min. Patients were then tilted upward at angle of 60° for 45 min. Then, blood pressure and heart rate were monitored and electrocardiogram was recorded simultaneously during the test (Dash 2000 Monitor, GE Company, U.S.A). The patients were placed in the supine from the standing position as soon as the positive response or symptoms of orthostatic intolerance (OI) occurred. The symptoms of OI included lightheadedness, diminished concentration, tremulousness, nausea, headache, near syncope or syncope. A sustained heart rate increase ≥ 40 beats/min or a sustained rate of 120 beats/min in the first 10 min of passive upright tilt is considered diagnostic [17,18].

### Assay orthostatic plasma norepinephrine

After patients rested for 30 min or longer, they remained standing position for 5 min, and then blood samples were collected. Blood samples were centrifuged at 3,000 rpm for 10 min immediately after collection, and plasma was frozen for −20°C until analysis. Plasma catecholamine levels were determined by high-performance liquid chromatography with electrochemical detection [19].

### Protocol of treatment and follow-up

All 27 patients were prescribed metoprolol for therapy. The dose of metoprolol was 0.5 mg/kg daily, twice a day. Follow-up in our cardiology clinic was conducted after 3 month of therapy, and the children and their parents were asked to report any changes in symptoms of orthostatic intolerance.

POTS symptoms were evaluated in children with the severity of symptoms for symptom scores [17,20,21]. Symptom score criteria: the following symptoms: dizziness, headache, shortness of breath, gastrointestinal symptoms, fatigue, pallor, blurred vision, palpitations, sweating, tremulousness, according to their frequency of OI symptoms graded from 0 to 4. Score 0 stands for no symptoms occurrence during follow up; score 1: 1 episode of OI symptom occurrence per month; score 2: 2 to 4 episodes of symptoms occurrence per month; score 3: 2 to 7 episodes of symptoms occurrence per week; 4: more often once daily. The therapy was determined to be effective, when the symptom of OI disappeared or symptoms scores decreased less than 50 percent [22].

### Statistical analysis

Statistical analysis was done using SPSS13.0 (SPSS Inc, Chicago, IL, USA). Continuous variables were compared using Student’s *t*-test and analysis of variance; categorical variables were compared using Fisher’s exact test, and qualitative variables were compared using Pearson’s chi-square test. A P value <0.05 was considered to be statistically significant. The relationship between symptom severity and increment of heat rate from supine position to standing with orthostatic plasma norepinephrine level in children with POTS was analyzed using the Pearson correlation test. A receiver-operating characteristic (ROC) curve was utilized to evaluate the predictive value of orthostatic plasma norepinephrine level in assessing the therapeutic effect of metoprolol. The area under the curve represents the predictive value, with an area of 0.5-0.7 indicating low predictive value, 0.7-0.9 indicating a moderate predictive value, and >0.9 indicating high predictive value. If the 95% CI of the area under the curve did not contain 0.5, or if the P value was <0.05, it was suggested that orthostatic plasma norepinephrine level was a reliable predictor of the therapeutic effect of metoprolol in treating children with POTS.

## Results

Mean age for all 27 POTS patients was 11 ± 6 years, ranging from 6 to 15 years. There was female predominance (females: males: 15:12). The mean BMI of 27 patients was 18.6 ± 3.7. Supine HR and BP were shown in Table 1.

**Table 1:** **Comparison of baseline characteristics in responders and nonresponders**

**Characteristics**	**Responders**	**Nonresponders**	**P value**
Cases (n = 25)*	13	12	
Age, years	12 ± 2	11 ± 3	0.655
BMI, kg/m2*	19.7 ± 4.9	17.2 ± 2.5	0.130
Females/males, n	7/6	6/6	0.716
Symptoms score before treatment	7.4 ± 1.9	7.2 ± 2.7	0.820
Supine systolic BP, mmHg	101.9 ± 9.3	98.0 ± 8.6	0.286
Supine diastolic BP, mmHg	58.0 ± 9.7	55.1 ± 8.1	0.426
Supine hear rate, beats/min	74.0 ± 9.4	75.6 ± 9.8	0.683
Orthostatic plasma norepinephrine level, pmol/ml	5.10 ± 2.69	2.93 ± 1.79	0.028

*Two out of 27 cases lost during follow-up.

**Figure 1:**
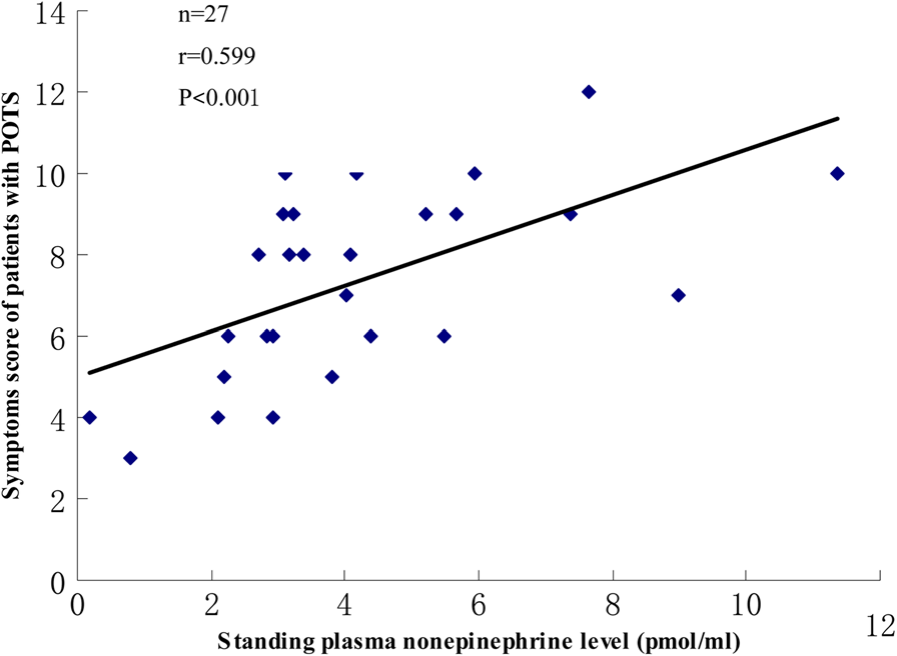
**Scatter plot and trend line of symptoms score and orthostatic plasma norepinephrine level of patients with POTS.**

**Figure 2:**
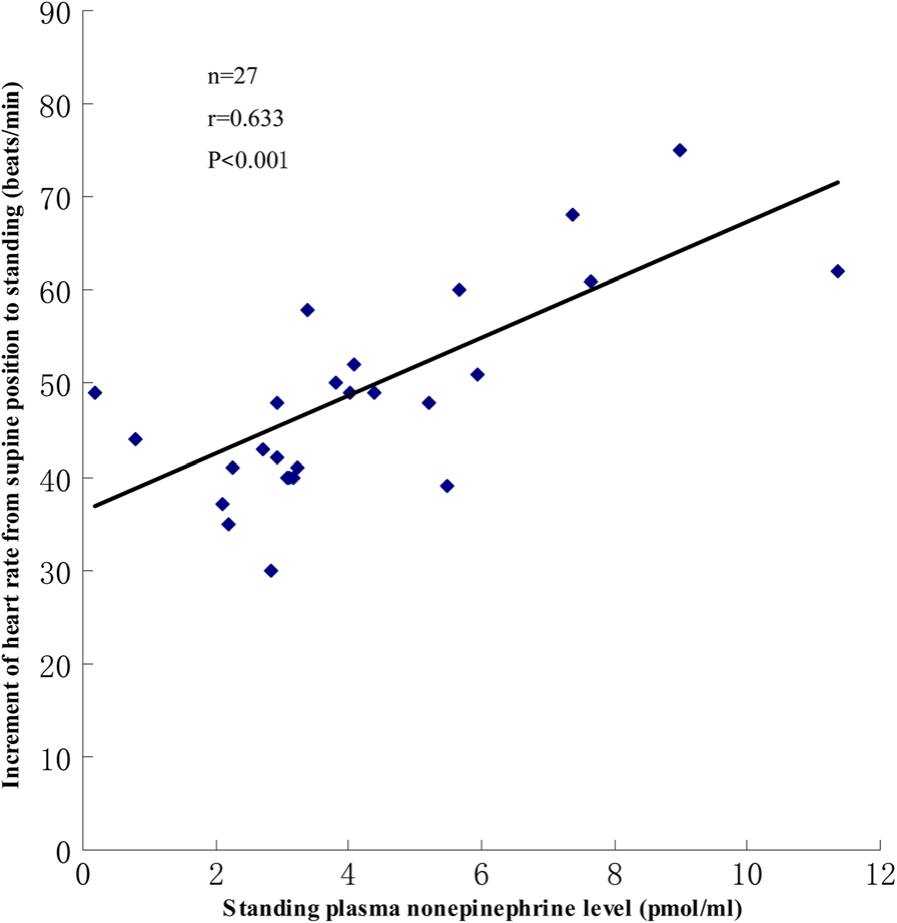
**Scatter plot and trend line of increment of heat rate from supine position to upright and orthostatic plasma norepinephrine level of patients with POTS.**

Patients with severe symptoms had a high level of orthostatic plasma norepinephrine. Pearson correlation analysis revealed a significant positive correlation between symptom severity and orthostatic plasma norepinephrine (r =0.599; P < 0.001) (Figure 1). Increment of heat rate from supine position to standing of patients with POTS also had a significantly positive correlation with orthostatic plasma norepinephrine through Pearson correlation analysis (r =0.633; P < 0.001) (Figure 2).

During follow-up period, 2 patients did not reply despite multiple attempts at making contact. Thus, we analyzed clinical symptoms and hemodynamic changes before and after metoprolol treatment in 25 patients. Both their symptom scores and their tachycardia during HUT or HUTT were decreased obviously, when compared with those before treatment (Table 2).

**Table 2:** **Comparisons of symptom scores, changes of heart rate in POTS children treated by metoprolol**

**Treatment**	**Responders (n = 13)**		**Nonresponders (n = 12)**	
	**Symptoms scores**	**Delta HR (beats/min)**	**Symptoms scores**	**Delta HR (beats/min)**
Pre-treatment	7.4 ± 1.9	55.2 ± 12.7	7.2 ± 2.7	43.6 ± 8.7
Post-treatment	0.4 ± 1.1	27.4 ± 7.8	5.0 ± 2.2	32.3 ± 9.7
P value	<0.001	<0.001	<0.001	0.001

In the 25 patients with POTS, who received metoprolol for therapy, 13 patients were responders, with their symptom disappearance or symptoms scores decrease less than 50 percent. The pre-treatment data, such as age, sex, symptom scores, supine blood pressure and changes in heart rates during HUT, did not differ between the 13 metoprolol responders and 12 nonresponders (Table 1).

**Figure 3:**
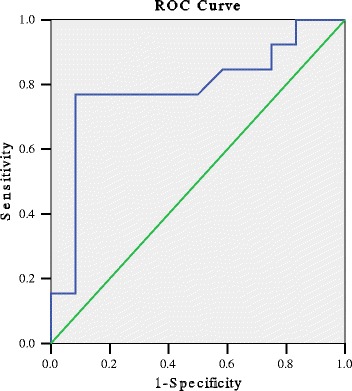
**ROC curve of predictive value of orthostatic plasma norepinephrine level for distinguishing responders and non-responders to metoprolol treatment.** The y-axis represents the sensitivity to predict the therapeutic response to metoprolol treatment in patients with POTS; the x-axis, the false positive rate (1 = specificity). Area under the curve was 0.785 (95% confidence interval: 0.591 to 0.980, P < 0.05).

The results showed that metoprolol responders had higher plasma levels of norepinephrine than the nonresponders, and the difference was obvious (P < 0.05; Table 1). The ROC curve (Figure 3) of plasma norepinephrine, for predicting the therapeutic effect of metoprolol, showed that the area under the ROC curve was 0.785 (95% confidence interval: 0.591 to 0.980, P < 0.05). Using a cutoff value for plasma levels of norepinephrine of 3.59 pg/ml (to convert to pg/mL, multiply by the molecular weight of norepinephrine) yielded a sensitivity of 76.9% and specificity of 91.7% in predicting the effect of metoprolol for treating POTS (Figure 3).

## Discussion

Our findings showed a clear association between the orthostatic plasma norepinephrine level and symptom severity in patients with POTS. The orthostatic plasma norepinephrine level of POTS patients also associated with the increment of heat rate from supine position to standing. Especially, we found that the effectiveness of metoprolol in POTS children was related to the level of orthostatic plasma norepinephrine. Our results indicate that plasma level of norepinephrine > 3.59 pg/ml is an indicator of the effectiveness of metoprolol in children and adolescents with POTS.

Children with POTS often have symptoms of OI, such as syncope, dizziness, chest distress, chest pain, headache, palpitation, fatigue, and so on [1–4]. Additionally, the recurrent symptoms usually create physical and psychological stresses in children’s daily lives, both at home and school [5,23,24]. Therefore, an effective treatment, to improve symptoms, is necessary for the children with POTS.

No definite cause of primary POTS has been identified [1–4]. Exaggerated postural tachycardia may reflect several pathophysiologically distinct mechanisms. Although the precise mechanism for POTS has not been determined, given that excessive tachycardia is a cardinal feature of this syndrome, a logical treatment strategy would be to reduce the HR with β-adrenergic blockade. Raj et al. reported that low-dose oral propranolol significantly attenuated tachycardia and improved symptoms in POTS, and higher-dose propranolol did not further improve, and might worsen symptoms [8]. Arnold et al. also found recently that propranolol could improve HR control and exercise capacity in POTS when given at the low doses of propranolol for treatment [10]. We also found that metoprolol plus oral saline was effective in the treatment of POTS in children [9]. But in experimental models of orthostatic intolerance, neither propranolol, nor esmolol was found to improve orthostatic tolerance [25–27], and Fu et al’ study suggested that for patients with POTS, propranolol was not superior to exercise training at restoring upright hemodynamics, normalizing renal-adrenal responsiveness, and improving quality of life [11]. The reasons for the different efficacy of β-blockers for treatment of POTS were differences of noradrenergic activity among distinct pathophysiological subtypes of POTS. Thieben et al. described 3 groups of patients: “hypovolemic”, “hyperadrenergic”, and “autoimmune” groups [28]. Clearly, not all cases of POTS are caused by hyperadrenergic state. Hyperadrenergic subgroup with β-blockers would seem reasonable. Thus, identifying an indicator to predict the effectiveness of β-blockers in these patients is important.

Increases in orthostatic plasma norepinephrin are the core of the biochemical changes of hyperadrenergic POTS patients. Thus, we hypothesized that orthostatic plasma norepinephrine level could predict the effectiveness of β-blockers in children and adolescents with POTS. In the present study, we found that higher orthostatic plasma norepinephrine level was associated with severer symptoms in children and adolescents with POTS, and there was also a positive correlation between heart rate increment on HUTT and orthostatic norepinephrine levels. These findings suggested that the excessive increase in orthostatic norepinephrine levels may result from impaired baroreflex-mediated vasoconstriction on standing in patients with POTS. These results are in keeping with previous studies of a prospective cohort of adult patients with POTS [29].

To examine the possible predictive value of plasma orthostatic norepinephrine levels, we used ROC analysis and showed that plasma levels of orthostatic norepinephrine had sensitivity of 76.9% and specificity of 91.7% in predicting the efficacy of metoprolol for treating POTS. Therefore, the plasma level of orthostatic norepinephrine could be taken as one of the reference indices in choosing medication for children with POTS.

The present study has limitations. The small sample size might have led to bias. Furthermore, we did not detect the orthostatic plasma norepinephrine level of healthy children as control group and the orthostatic plasma norepinephrine levels after the treatment of metoprolol, which was the limitation of the study. Further studies are needed to increase the case number in the multi-center design and analyze the plasma norepinephrine levels in a more comprehensive aspect. It is known that hyperadrenergic state has been well recognized one of the important mechanisms for POTS, and present study firstly provided the information about the role of plasma norepinephrine as a biomarker to predict the effect of treatment of metoprolol on POTS children.

## Conclusion

Our findings suggest that the orthostatic plasma norepinephrine level could be of great help in selecting metoprolol therapy in the management of POTS in children and adolescents. The findings are of great importance for the individual therapeutic strategy of POTS in children and adolescents.

## Abbreviations

POTS: Postural tachycardia syndrome
ROC: Receiver-operating characteristic
HUT: Head-up test
HUTT: Head-up tilt test
OI: Orthostatic intolerance
CI: Confidence interval
